# Dry extract BNO 1011 inhibits human influenza a replication and neuraminidase activity in oseltamivir-resistant and -sensitive viral strains

**DOI:** 10.1186/2045-7022-3-S2-P20

**Published:** 2013-07-16

**Authors:** Stephanie Seifert, Katja Wosikowski, Jutta Haunschild

**Affiliations:** 1 Bionorica SE, Preclinical and Project Management (R&D), Neumarkt i.d.Opf., Germany

## Background

Virus infection is the main triggering event for the development of acute rhinosinusitis and human influenza A virus ranks among the most frequent viral causes of infection. Influenza neuraminidase, a key enzyme in viral replication, spread, and pathogenesis, is the primary target in prevention and treatment of influenza infection. Sinupret^®^, a herbal medicinal product composed of Gentianae radix, Primulae flos, Sambuci flos, Rumicis herba, and Verbenae herba, is frequently used for the treatment of acute rhinosinusitis.

## Objective

To investigate the anti-viral activity of the Sinupret^®^ dry extract BNO 1011 *in vitro* and its potential to inhibit neuraminidase in oseltamivir-resistant and -sensitive human influenza A H1N1 strains of clinical relevance.

## Methodology

*In vitro*, BNO 1011 was tested for its interference with human influenza A infection using a plaque reduction assay in MDCK cells. The impact on two clinically relevant human influenza A H1N1 strains displaying divergent sensitivity against the well-known neuraminidase inhibitor oseltamivir (OS) was studied (OS-sensitive: human influenza A/California/07/2009; OS-resistant: human influenza A/Maryland/04/2011). In addition, BNO 1011 was studied for its inhibitory activity on neuraminidase of the same influenza A strains in a highly sensitive chemiluminescence assay. The *in vitro* experiments were paralleled by monitoring viability of MDCK cells in the presence of BNO 1011.

## Results and conclusions

BNO 1011 efficiently blocked the infectivity of both influenza A H1N1 strains studied in a plaque reduction assay [EC_50_: 8.3 µg/mL (A/California/07/2009), 8.2 µg/mL (A/Maryland/04/2011)] but did not affect cell viability (CC_50_>1 mg/mL), yielding a therapeutic index (CC_50_/EC_50_) of 121 and 122, respectively. BNO 1011 also inhibited neuraminidase activity in both viral strains with comparable efficiency [IC_50_: 59.3 µg/mL (A/California/07/2009), 99.7 µg/mL (A/Maryland/04/2011)], irrespective of the strains' oseltamivir sensitivity. Taken together, Sinupret^®^ dry extract BNO 1011 inhibits the replication of clinically relevant influenza A isolates. As underlying mechanism, the inhibition of viral neuraminidase was identified. Our results support the application of Sinupret^®^ dry extract BNO 1011 in the treatment of acute, viral rhinosinusitis.

